# Spatial mapping of HIV testing and antiretroviral therapy initiation uptake among the adolescents and youth in rural KwaZulu-Natal, South Africa

**DOI:** 10.1080/09540121.2025.2611339

**Published:** 2026-03-16

**Authors:** Mulugeta Shegaze Shimbre, Andrew Gibbs, Nondumiso Mthiyane, Jaco Dreyer, Natsayi Chimbindi, Nigel Garrett, Andrew Tomita, Maryam Shahmanesh

**Affiliations:** a Discipline of Public Health Medicine, School of Nursing and Public Health, University of KwaZulu-Natal, Durban, South Africa; b Health Economics and HIV and AIDS Research Division (HEARD), University of KwaZulu-Natal, Durban, South Africa; c Department of Epidemiology, School of Public Health, College of Medicine and Health Sciences, Arba Minch University, Arba Minch, Ethiopia; d Department of Psychology, University of Exeter, Exeter, United Kingdom; e Gender and Health Research Unit, South African Medical Research Council, Pretoria, South Africa; f Centre for Rural Health, School of Nursing and Public Health, University of KwaZulu-Natal, Durban, South Africa; g Africa Health Research Institute, Durban, South Africa; h Institute for Global Health, University College London, London, UK; i The Desmond Tutu HIV Centre, University of Cape Town, Cape Town, South Africa; j Centre for the AIDS Programme of Research in South Africa (CAPRISA), University of KwaZulu-Natal, South Africa; k KwaZulu-Natal Research Innovation and Sequencing Platform (KRISP), College of Health Sciences, University of KwaZulu-Natal, Durban, South Africa; l School of Medicine, University of KwaZulu-Natal, Durban, South Africa

**Keywords:** Adolescents, HIV, South, Africa, Good health and well being

## Abstract

This study investigated the spatial distribution of HIV testing and antiretroviral therapy initiation among adolescents and youth in rural KwaZulu-Natal, South Africa, to inform the optimal allocation of HIV services. The sample included 5,352 participants aged 13–35 years from nested cohort studies within Africa’s largest population-based prospective cohorts, with global positioning system data collected for all homesteads. HIV testing prevalence and ART initiation prevalence were mapped using a kernel density approach, and socio-demographic correlates were examined using multivariable regression. Kernel density mapping revealed substantial geographic disparities in HIV testing and ART initiation. Multivariable analysis showed that tertiary education (aβ: 4.53; 95% CI: 2.11, 6.88), rural residence (aβ: −31.14; 95% CI: −32.32, −29.65), and living within 3 km of a health facility (aβ: −2.42; 95% CI: −3.88, −0.96) were associated with HIV testing prevalence. ART initiation prevalence was associated with rural residence (aβ: −5.44; 95% CI: −7.38, −3.37), participation in HIV self-testing (aβ: 3.21; 95% CI: 0.64, 5.80), and lack of participation in STI screening and treatment (aβ: −1.22; 95% CI: −2.44, −0.03). Our research underscores the urgent need for addressing geographical access to HIV testing and treatment uptake amongst adolescents and youth in rural communities, and promoting HIV self-testing.

## Introduction

HIV remains a significant global health challenge, with an estimated 65 – 113 million infections and 32.9 – 51.3 million deaths over the past 40 years (Chimbindi et al., 2020). The 2024 report of the Joint United Nations Program on HIV/AIDS (UNAIDS) showed that global progress against new HIV acquisitions was slowing rather than accelerating, with a decrease of 3.62%, being the smallest annual reduction since 2010 (UNAIDS. FACT SHEET, 2024). In sub-Saharan Africa (SSA), while the HIV incidence has decreased, disparities persist (Birdthistle et al., 2018), such as HIV prevalence among adolescents in South Africa increased from 3.0% in 2012–3.7% in 2017 (Naidoo et al., 2022). For adolescent girls and young women, HIV incidence was 3.92 per 100 person-years (Lewis et al., 2022). Poor access to HIV testing and treatment can result in delayed diagnoses, inconsistent treatment adherence, and higher rates of HIV-related complications, especially in underserved communities (Birdthistle et al., 2018).

Antiretroviral therapy (ART) has significantly improved life expectancy for people living with HIV (PLWH), bringing it in line with that of the general population in most countries (Gourlay et al., 2019). However, a considerable number of individuals remain undiagnosed or unaware of their HIV status, which poses a barrier to effective treatment and care. UNAIDS has set ambitious “95-95-95” targets for 2025, aiming for 95% of PLWHs to know their status, 95% of those diagnosed as living with HIV to receive ART, and 95% of those on ART to achieve viral suppression (Mthiyane et al., 2022). Achieving the first 95% (HIV testing) is essential for linking individuals with an HIV-positive status to appropriate care, this remains a challenge in SSA, where HIV testing uptake remains variable across settings, particularly in rural areas, where geographic distance, transportation barriers, and associated costs limit access (Ante-Testard et al., 2023; Baisley et al., 2019; Gareta et al., 2021; Kharsany et al., 2018). Awareness of HIV-positive status in East and Southern African nations ranges from 58% to 87%, with adolescents and youth often being less likely to have been tested (Nkambule et al., 2023). A study conducted in South Africa indicated that the level of HIV testing among districts ranged from 54.7% to 86.1%, indicating a possible disparity in the provision of services and care uptake. KwaZulu-Natal (KZN) Province has the widest variations in HIV testing coverage across South Africa, with the lowest uptake reported in the northern rural uMkhanyakude District (54.7%) (Jooste et al., 2021b).

HIV testing disparities in South Africa are influenced by various socio-demographic factors, including geographical variations (Zuma et al., 2022), with urban men and women more likely to test for HIV than those living in rural informal settlements, which reflects the inequalities in access to resources and healthcare services (Baisley et al., 2019). Men experience worse HIV-related outcomes, with lower rates of status awareness and treatment uptake than women (Shapiro et al., 2020; Sharma et al., 2019). Additionally, young age, lower socio-economic status, and education levels, unemployment, alcohol consumers in low or poor socio-economic status (Jooste et al., 2020; Jooste et al., 2021a; Tshivhase et al., 2022), fear of positive results (De Wet & Kagee, 2018), stigma (De Wet & Kagee, 2018; Tshivhase et al., 2022), and long distances to health facilities are associated with reduced testing rates (De Wet & Kagee, 2018; Tshivhase et al., 2022).

Despite policies like Universal Test and Treat (UTT) and Same-Day Initiation (SDI) being in place since 2017, concerns persist regarding the low rate of ART initiation (Shapiro et al., 2020), with many individuals knowing that they are living with HIV, delaying treatment initiation. In South Africa, ART initiation varies significantly across districts (Jooste et al., 2020; Jooste et al., 2021a; Larsen et al., 2019), with PLWH in rural areas often facing greater challenges in accessing HIV care services than those in urban settings (De Wet & Kagee, 2018; Tshivhase et al., 2022). The variation is due to variations in political leadership, program design, monitoring systems, and external partnerships, with substantial differences in treatment access (Jooste et al., 2020; Jooste et al., 2021a; Larsen et al., 2019). Recent studies in KwaZulu-Natal, South Africa, have shown varying rates of linkage to HIV care and ART initiation. A study reported a 62% linkage rate within 3 months (Nicol et al., 2023a), while another found rates as high as 83% in the uMgungundlovu district (Nicol et al., 2023b).

Factors affecting ART initiation include age, with adolescents and youth PLWHs being less likely to initiate treatment, as emphasized within the “test and treat” framework (Iwuji et al., 2018; Sy et al., 2021). In addition, lack of time, a desire to delay treatment until illness onset, fear of side effects, disbelief in the HIV diagnosis (Maughan-Brown et al., 2021), and limited access to ART (Larsen et al., 2019; Loeliger et al., 2016), particularly among those residing in rural areas as well as regions affected by violence (Loeliger et al., 2016), can impact timely ART initiation. Furthermore, insufficient patient education, social support, and dissatisfaction with healthcare services are key factors affecting HIV testing and ART initiation (Loeliger et al., 2016). Apart from reporting different rates of HIV testing and ART initiation, there are also inconsistent factors associated with the reasons for both. Overall, these differences affect the fight against HIV, as they hinder early detection and timely treatment initiation, leading to increased transmission rates and poorer health outcomes. This study, therefore, aimed to undertake a secondary analysis by mapping the distribution of HIV testing and initiation of ART among adolescents and youth aged 13 – 35 in the high HIV-prevalence and under-resourced uMkhanyakude District in KZN, South Africa.

## Methods and materials

### Study design

As part of the impact evaluation of a multicomponent HIV prevention intervention for adolescent girls and young women (AGYW) in the uMkhanyakude district of KwaZulu-Natal, we recruited and followed up two population-representative groups of adolescents and youth randomly selected from the health and demographic surveillance survey (HDSS) between 2017 and 2019 (Chimbindi et al., 2018; Chimbindi et al., 2020). The groups were followed up on yearly for one to two years until 2019 (Birdthistle et al., 2018). Here, we present a cross-sectional analysis of the baseline surveys in these two groups to establish the distribution of those who tested for HIV and started ART. The two groups were involved in the analysis from a nested cohort 1 and nested cohort 2 studies. The nested cohort 1 study focused on adolescent girls and young women, while the nested cohort 2 study included adolescents and youth adults of both sexes.

### Setting and population

The uMkhanyakude District is characterized by poverty, with high prevalence rates of HIV (36.3%) (Kharsany et al., 2018), low ART coverage of 56% - 70%, and where approximately 85% of young people are jobless (Gareta et al., 2021). This study included male and female adolescents and youth participants aged 13–35 from both studies to spatially map HIV testing uptake and ART initiation.

A sample of AGYW aged between 13–22 years was randomly selected from a list of age-eligible residents recorded in the HDSS in 2017. A random sample was stratified by age (13–17 and 18–22) and by 45 geographic areas (nested cohort 1). Similarly, a random stratified sample of adolescent boys and young men (ABYM) aged 13–35 and young women (YW) aged 24–29 was selected in 2018. A sample was stratified by age (13–17, 18–24, 25–29, 30–35), gender (male and female), and 45 geographic areas (nested cohort 2).

**Figure 1.**
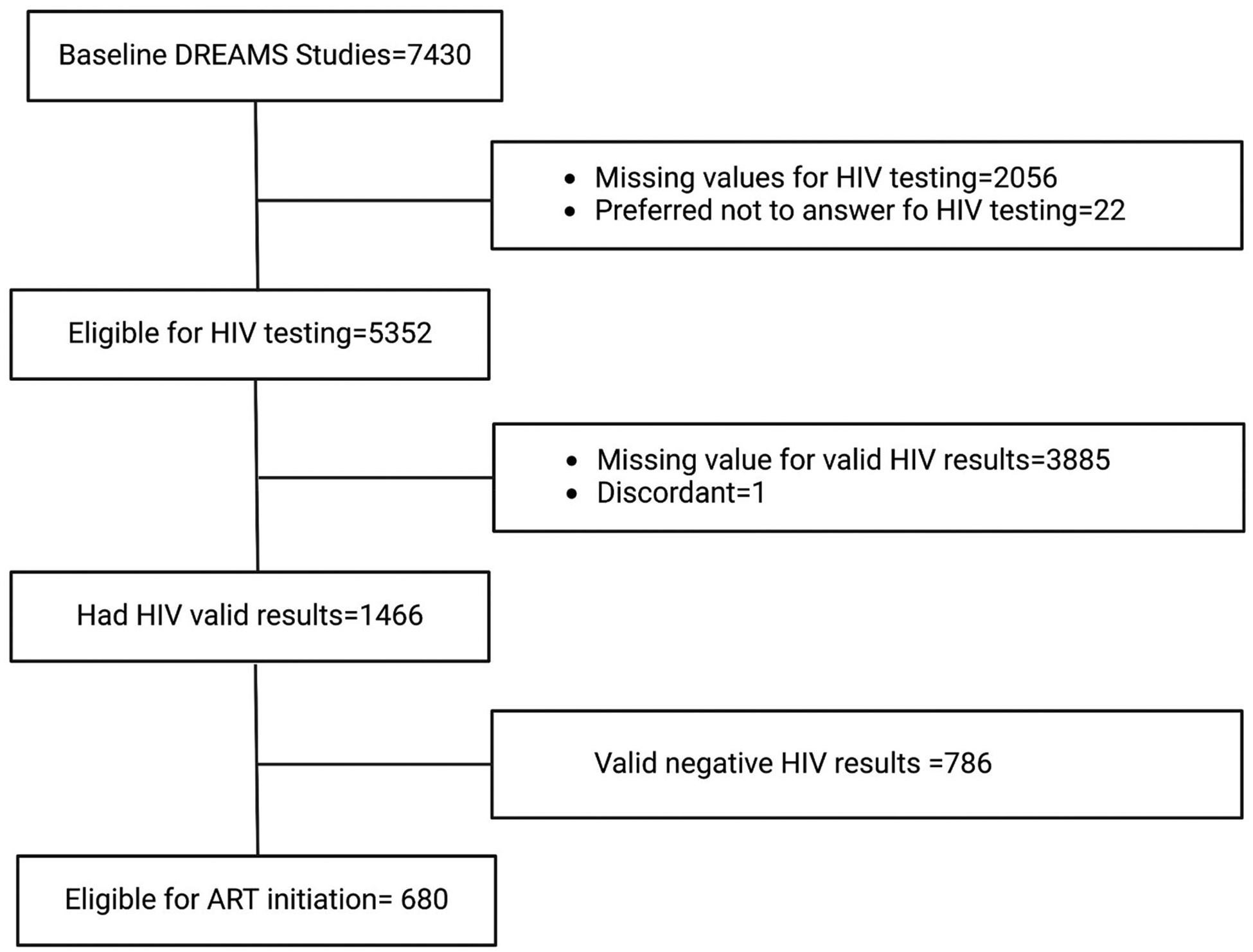
Flow chart of participant inclusion into secondary data analysis from the parent studies datasets.

The sampling frames of the groups, comprising 7,430 participants (3,010 aged 13–24 from the nested cohort 1 and 4,420 aged 13–35 from nested cohort 2), were included in the baseline assessment. As this study focused on HIV testing and ART initiation, the sample population for analysis was limited to those who self-reported having taken an HIV test in the baseline survey at enrollment into the two groups. Based on that restriction, the sample totaled 5,352 participants (3,060 (57.17%) from nested cohort 2 and 2,292 (42.83%) from nested cohort 1) (Figure 1).

The Africa Health Research Institute(AHRI) (Gareta et al., 2021) demographic and surveillance program collected geospatial information (latitude and longitude coordinates) through the use of global positioning systems (GPS) for all homesteads in the study area, with each homestead assigned a unique identifier. This coding system enables individuals to be assigned to their respective homesteads, allowing accurate mapping of their demographic details, thereby facilitating the spatial analysis of HIV testing and ART initiation.

### Data collection and management

Data were obtained from two databases that included the two groups. The groups utilized both self and interviewer-administered tablet-based interview methods for collecting data from assenting/consenting study participants in isiZulu, the local African language, and English. For sensitive topics, such as sexual behavior and violence, study participants were provided with a tablet to conduct a self-administered interview.

For certain clinical/hospital treatment data, the group obtained data from the TIER.net system, with permission from study participants. TIER.net is an electronic system used for monitoring and evaluating HIV care and treatment programs at government health institutions throughout South Africa (Baisley et al., 2019; Birdthistle et al., 2018), including facilities covered in the AHRI catchment area. Although pre-ART visits are not recorded in TIER.net, the database contains data on the attendance of clinic visits, laboratory test results, and the records of dispensing ART for the majority of patients receiving treatment.

Explanatory analysis was conducted to verify completeness, the missing values being assessed by comparing all dataset variables with the study questionnaires. Outcome variables with alternative responses, such as 9 responses recorded as “preferred not to answer,” were changed to missing values. In the case of independent variables, missing responses were documented but kept unless proven analytically insignificant. Unmatched and missing values were identified and resolved during dataset merging or appending to uphold data quality. This approach ensured that missing data were handled systematically to reduce bias and preserve the reliability of the analysis.

## Outcome measurement

The first study outcome is HIV testing prevalence (based on continuous measures). The outcome variable was operationalized based on individual HIV testing (binary measure) based on self-reports from nested cohort 1 and nested cohort 2 datasets. Data on actual HIV testing were not available in the dataset. Each individual who was assessed for ever-have HIV testing in the AHRI demographic surveillance system linked individuals to their respective bounded structure using GPS. Toward estimating HIV testing prevalence, we totaled the number of individuals who had HIV testing cases divided by the number of individuals residing in a given geographic point (i.e., GPS coordinates). The second outcome is ART initiation prevalence (based on continuous measures). The outcome variable was operationalized based on ART initiation data from the Tier.net dataset. The ART initiation prevalence is generated by the total number of individuals who initiated ART by count divided by the total number of individuals who had valid HIV-positive results in a given geographic point (i.e., GPS coordinate).

## Independent variables

The independent variables were chosen based on their relevance to the study outcomes, specifically HIV testing and ART initiation. The selection process involved an explanatory analysis to verify data completeness and assess missing values by comparing dataset variables with study questionnaires. The independent variables included age, gender, level of education, residence, participation in facility-based HIV testing, counseling before the last HIV testing, participation in HIV self-testing, participation in STI screening and treatment, referral to treatment, distance to health facilities, and social media exposure. The datasets used for this analysis included nested cohort 1and nested cohort 2. These variables were documented and retained unless proven analytically insignificant during the dataset merging or appending process to ensure data quality and reliability.

### Analysis

Three analyses were conducted, the first being summarizing sociodemographic and clinical characteristics of study participants in the current investigation. Frequencies and percentages were calculated for categorical variables, and means with standard deviation (SD) were used for continuous variables.

Second, to map the distribution of HIV testing prevalence and ART initiation prevalence, we applied a kernel density approach using ArcGIS version 10.4 (Waller & Gotway, 2004). By applying a kernel function, which assigns weights to nearby data points based on their distance, kernel density mapping generates a smooth surface that highlights areas of higher and lower concentrations of individuals tested for HIV and those who initiated ART. This method also avoids imposing rigid geographical boundaries on the data and produces maps of the distribution of HIV test prevalence and ART initiation prevalence estimates that are responsive to local variations while remaining resilient against random fluctuations (Tanser et al., 2013).

In this current study, a kernel density (quintile method) with a radius of 3 km was applied to the grid (Rossi et al., 1992; Tanser et al., 2009) to systematically measure the spatial distribution of and create plots to visualize the geographic distribution of their HIV testing prevalence. Each grid cell represents a localized area, with the residence of every HIV-tested individual geo-located within it. These data points were used to assess HIV testing prevalence, defined as the proportion of individuals within each grid cell who have tested for HIV. Likewise, a kernel density was applied to systematically measure the spatial distribution of and create plots to visualize the geographical distribution of their ART initiation prevalence.

Third, we fitted linear regression models (both bivariate and multivariable) using STATA version 18 (StataCorp, L. L. C, 2017) to identify factors associated with HIV testing prevalence and initiation of ART prevalence. R^2^, the proportion of variance in the outcome variable that’s explained by the independent variables, will be reported for the multivariable model. To evaluate multicollinearity, only variables with a variance inflation factor (VIF) less than 5 were included in the regression models. A *p*-value <0.05 was considered statistically significant, with confidence intervals (CIs) also being reported.

## Results

### Participants’ sociodemographic and clinical characteristics

A total of 5,352 participants were assessed for HIV testing in the uMkhanyakude District, KZN; their median age was 19, with Interquartile Range (IQR) of 16–24, of whom 2,868 (53.59%) were females, 833 (38.58%) had some secondary education (grade 9–12) or below, while only 219 (10.14%) had tertiary education, and 96.92% reported living in rural areas (Table 1).

Among the 4,666 who answered the facility-based HIV testing question, 46.68% had taken up facility-based HIV testing. Counseling before HIV testing was received by 90.55% (n = 3641), while among the 3,540 respondents who answered a question about HIV self-testing (HIVST), only 4.49% reported having been offered and accepted HIV self-testing. Among the 4,168 participants who responded to STI screening and treatment, only 12.55% had received STI screening and/or treatment. Of the 5,300 participants who were asked about the distance to the nearest health facility, 45.68% (n = 2421) reported living less than 3 km away, while 36.98% (n = 1960) lived more than 5 km away. Out of the 3,060 participants assessed for media exposure, 76.21% used any social media (Table 1).

Table 1 also presents the baseline characteristics of 680 participants assessed for ART initiation; their median age was 25, with an IRQ of 20–28, and the majority (66.32%) being female. Among the 492 participants who reported their level of education, only 7.32% had a tertiary level, and of the 680, the majority resided in rural areas (96.17%). Among the 632 participants who answered the facility-based HIV testing question, 53.8% utilized this method, while 5.87% (n = 30) of 511 individuals used HIV self-testing. Of the 609 participants who answered the counseling before HIV testing question, 93.43% had pre-HIV test counseling during their last HIV test, and of the 571 who responded to STI screening and treatment, 24.17% reported receiving it. Out of the 672 participants who answered questions about the distance to the nearest health facility, 29.32% live further than 5 kilometers, and of the 444 participants who answered a social media usage question, 78.83% reported being exposed to social media.

### Kernel density mapping of HIV testing and ART initiation

**Figure 2.**
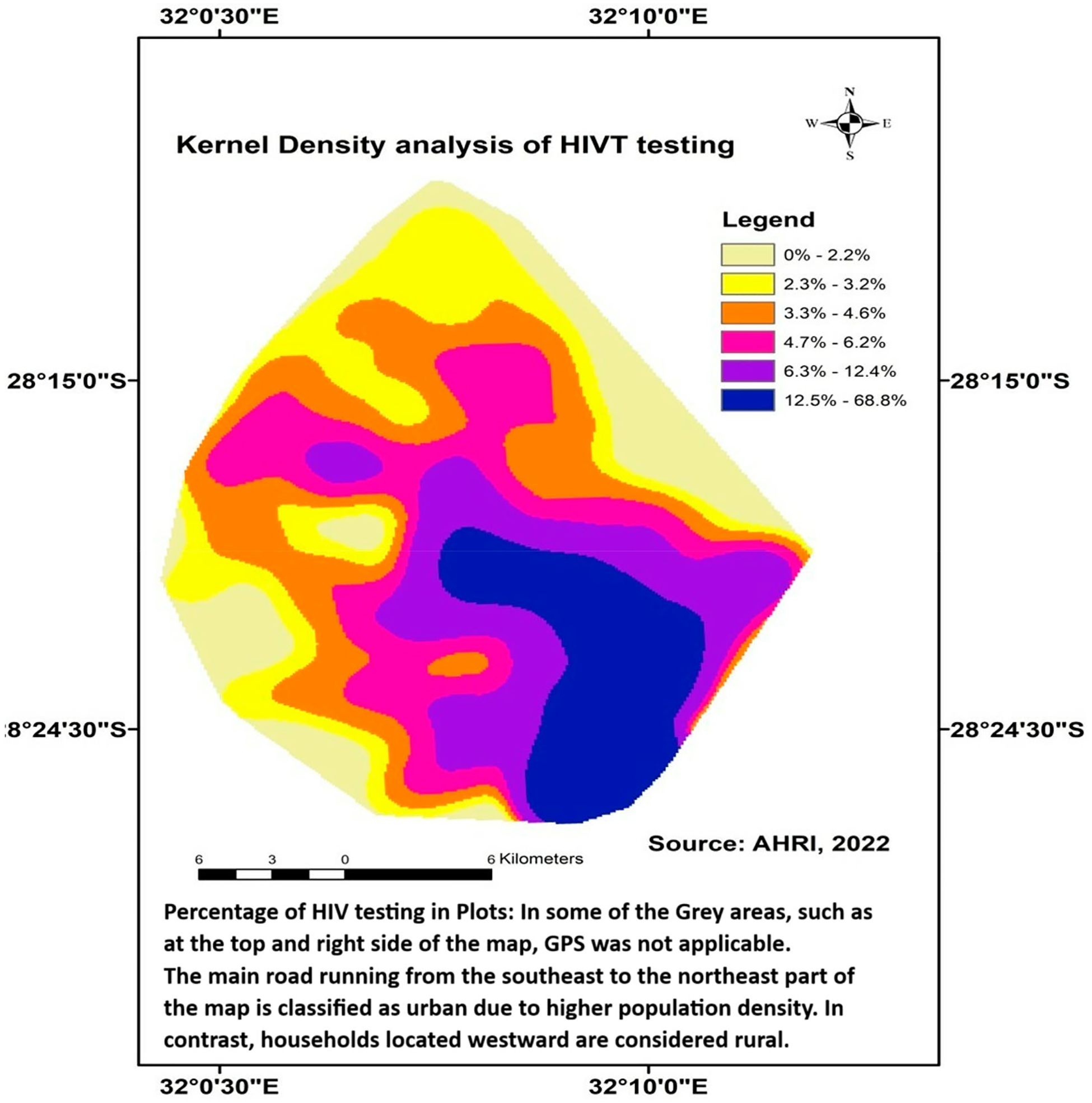
A kernel density analysis of the distribution of HIV testing prevalence among adolescents and youth in the uMkhanyakude District from 2017 to 2019.

The kernel density distribution map in Figure 2 illustrates HIV testing, showing the spatial distribution of individuals who knew their HIV status. The density plot compares the density of participants tested for HIV within each area to the total population with HIV tested. Darker Blue-shaded areas represent a higher density distribution of HIV testing, constituting 12.5%−68.8% of the total; the purple represents the intermediate high-density distribution, constituting 6.3%−12.4%; while darker white shades indicate less dense distribution ranges (0 – 2.2%).

**Figure 3.**
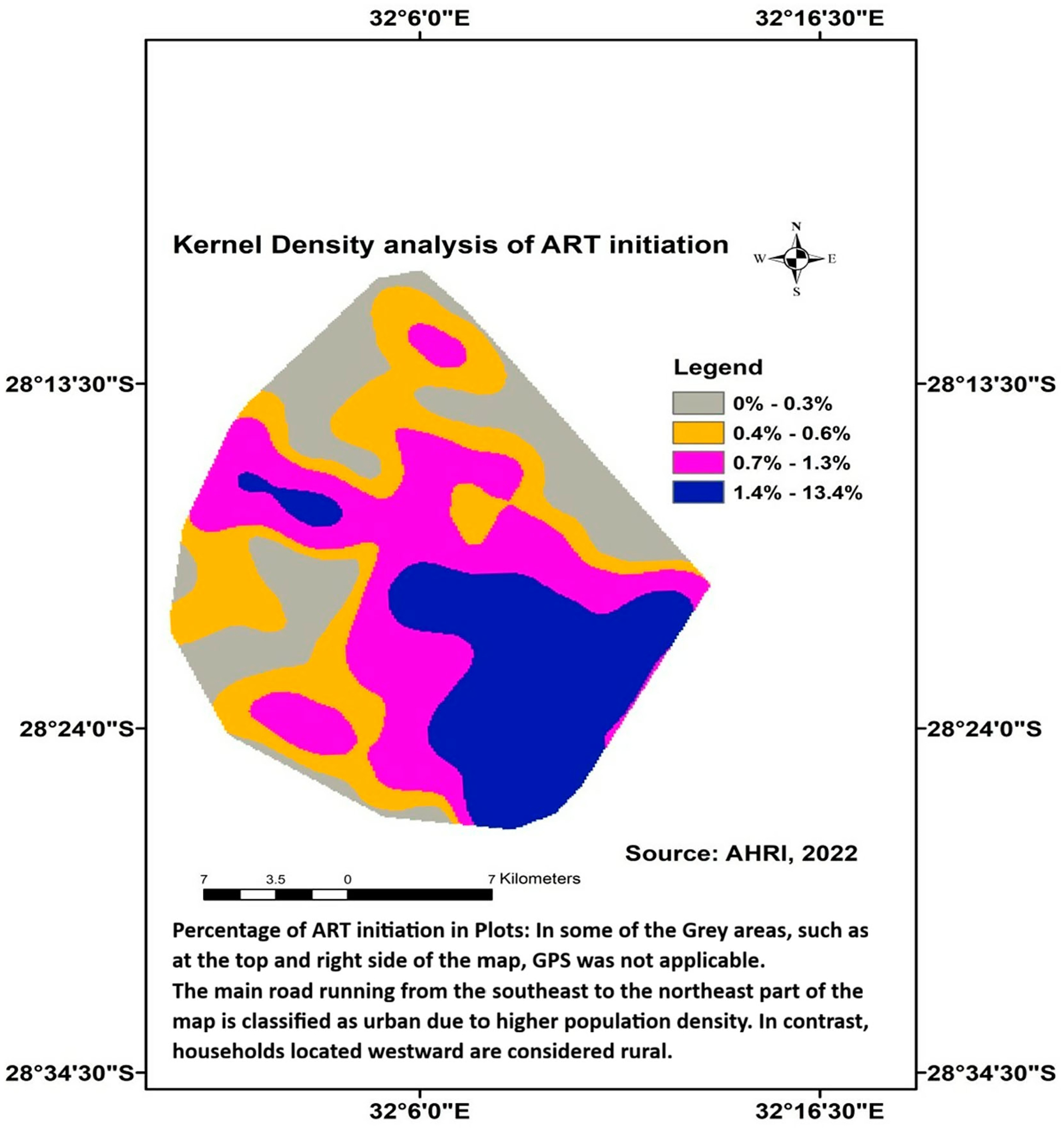
The kernel density analysis of ART initiation prevalence among adolescents and youth in the uMkhanyakude District from 2017 to 2019.

The Kernel density distribution map (Figure 3) illustrates the spatial distribution of individuals who had started ART, with the density plot highlighting the concentration of those in various plots among the overall participants who initiated. Darker Blue-shaded areas represent the highest densities of ART initiation, constituting 1.4% - 13.4% of the total; the purple-shaded areas indicate intermediate high-density distributions, accounting for 0.7%−1.3% of the total, while the lighter white shades correspond to the lowest density distribution ranges of 0 – 0.3%.

The disparity between the prevalence of HIV testing and ART initiation, as illustrated in Figures 2 and 3, highlights a critical gap in the continuum of HIV care. The self-reported HIV testing in the yellow plot in Figure 2 is higher than ART initiation in the yellow plot in Figure 3. This shows that a significant number of individuals who HIV tested and received a positive result did not initiate ART, which is essential for managing the virus and improving adolescent and youth health outcomes.

### Associated factors of HIV testing prevalence and initiation of ART prevalence

The linear regression analysis was used to determine the association between independent variables with HIV testing prevalence and ART initiation prevalence.

In the bivariate analysis for the first outcome variable (HIV testing prevalence), age, gender, education, residence, participation in STI screening and treatment, and distance to the nearest health facility were associated with HIV testing prevalence. However, in the multivariable analysis, only education, residence, participation in STI screening and treatment, and distance to the nearest health facility were significantly associated with HIV testing prevalence.

In the bivariate analysis for the second outcome variable(ART initiation prevalence), residence, participation in HIV self-testing, participation in STI screening and treatment, and distance to the nearest health facility were associated with ART initiation prevalence. Similarly, in the multivariable analysis a residence, participation in HIV self-testing, participation in STI screening and treatment, and distance to the nearest health facility were significantly associated with ART initiation prevalence.

Tertiary-level education attainment, compared to lower levels of education, is significantly associated with higher HIV testing prevalence (aβ = 4.53, 95% CI: 2.11, 6.88). Residing in rural areas, compared to urban areas, is significantly associated with lower HIV testing prevalence (aβ = −31.14, 95% CI: −32.32, −29.65). Living within 3 km of the nearest health facility, compared to living more than 5 km away, is significantly associated with lower HIV testing prevalence (aβ = −2.42, 95% CI: −3.88, −0.96). However, living within 3–5 km of the nearest health facility, compared to living more than 5 km away, is significantly associated with higher HIV testing prevalence (aβ = 2.11, 95% CI: 0.16, 3.88) (Table 2).

Regarding the ART initiation prevalence, residing in rural areas, compared to urban areas, is significantly associated with lower ART initiation prevalence (aβ = −5.44, 95% CI: −7.38, −3.37). Self-testing for HIV, compared to not self-testing, is significantly associated with greater ART initiation prevalence (aβ = 3.21, 95% CI: 0.64, 5.80). Non participation in STI services programs, compared to Participation, is significantly associated with lower ART initiation prevalence (aβ = −1.22, 95% CI: −2.44, −0.03) (Table 3).

## Discussion

The study aimed to undertake a secondary analysis by mapping the distribution of HIV testing and initiation of ART among adolescents and youth aged 13 – 35 in the high HIV-prevalence and under-resourced uMkhanyakude District in KZN, South Africa. Geographic analysis using kernel density mapping revealed that certain areas within the district experienced lower rates of HIV testing and ART initiation, highlighting significant spatial disparities in the uptake of these services, this study aimed to identify factors influencing the resulting distribution.

The results identified a wide range of HIV testing prevalence within the district, with some areas reporting coverage above 68% for individuals ever tested for HIV, while others fall below 13%. This range is wider than the findings from a cross-sectional study conducted in South Africa (Jooste et al., 2021b), which reported HIV testing uptake rates ranging from 54.7% to 85% across various districts. Similarly, a study in SSA countries reported regional variations in HIV testing (Ante-Testard et al., 2023; Lealem et al., 2024) but with a narrower range than our findings. Additionally, certain areas within the district have the lowest HIV testing prevalence, in line with a study finding from South Africa (Jooste et al., 2021b), that uMkhanyakude has the lowest coverage for both males and females. The study also found a low rate of ART initiation in the district, ranging from 0.3% to 13.4%, this being significantly lower than the rates reported in a study conducted in South Africa, which varied between 50.8% in the North West and 72.7% in Northern Cape Provinces (Johnson et al., 2017). A study conducted in Uganda (Chamla et al., 2007) found that the uneven distribution of ART sites across districts and counties led to ART initiation rates ranging from 20% to 42.9%. Similarly, a cohort study conducted in South Africa (Larsen et al., 2019) reported substantial district-level variability in ART initiation rates. These findings highlight the need for targeted interventions to improve HIV testing and ART initiation in the uMkhanyakude District, as addressing these disparities is important to ensuring better health outcomes for adolescents and youth living with HIV in the region.

This study revealed a notable gap between the prevalence of HIV testing and the initiation of ART among adolescents and youth in the uMkhanyakude District. Although HIV testing rates are relatively high, a significant number of those diagnosed with HIV do not start ART. Geographically, our findings suggest that lower ART initiation rates are concentrated in more rural areas, while urban areas tend to have higher uptake. Several studies also reported the discrepancy and showed increasing HIV status knowledge among adolescents and youth (Hornschuh et al., 2019; Shanaube et al., 2021). A study reported that about one-third of individuals presented for ART initiation (Sithole et al., 2021). This finding underscores the need for targeted interventions to improve HIV testing and ART initiation among adolescents and youth in the uMkhanyakude District.

The study found key factors contributing to local variations in HIV testing and ART initiation rates. The findings suggest that several factors are significantly associated with a lower rate of HIV testing, including education level below secondary school, rural residence, distance from health facilities while residing in a rural area, and not participating in HIVST, STI screening, and treatment programs hindered ART initiation. Residing in rural areas was associated with low rates of both HIV testing and ART initiation, this finding aligning with previous studies, indicating that adolescents and youth in rural settings are less likely to undergo HIV testing and begin ART (Adugna & Worku, 2022; Ajayi et al., 2020; Exavery et al., 2020; Jooste et al., 2021b; Magadi, 2017; Magadi & Gazimbi, 2017; Somefun et al., 2019). Previous studies suggest that this disparity is primarily due to constrained resources, which limit the availability of essential services to populations when and where they are needed (Kim et al., 2021; Shamu et al., 2019). In South Africa, factors such as limited education, inadequate social support, and dissatisfaction with healthcare services have been found to contribute to low HIV testing rates and delayed initiation of ART (Jooste et al., 2020; Loeliger et al., 2016), with a perceived low risk of HIV infection may further discouraging timely initiation (Govere et al., 2021).

Our findings indicate that closer proximity to a health facility is negatively associated with HIV testing but not with ART initiation, and are aligned with studies from South Africa that have reported that some PLWH may travel longer distances for testing to maintain anonymity and reduce stigma (Larsen et al., 2019; Mee et al., 2020). Similarly, research on gay, bisexual, and men who have sex with men (GBMSM) in Ghana has shown that stigma and privacy concerns contribute to a preference for more distant health facilities (Zigah et al., 2023). In Burkina Faso, HIV-positive women also travel significant distances to access prevention of mother-to-child transmission services, challenging the assumption that proximity enhances attendance (Nikiema et al., 2019). However, these findings contrast with studies (Chen et al., 2017; Paulin et al., 2015) that report a strong negative association between distance from healthcare facilities and HIV testing uptake, the result possibly being due to the lack of privacy (Chen et al., 2017; Tanser et al., 2021) and quality healthcare service (McLaren et al., 2014). In addition, individuals may prefer accessing facility-based services farther from their home to avoid stigma or being recognized by community members (Mee et al., 2020). Moreover, with expanded access to decentralized testing options, facility proximity may no longer be a meaningful proxy for accessibility (Meehan et al., 2018).

HIVST is emerging as a complementary approach to traditional HIV counseling and testing (HCT) services, with the potential to facilitate the initiation of ART, and while the relationship between them is complex (Harichund et al., 2019), our study demonstrates a positive association. This finding contrasts with several studies that report mixed results regarding the impact of HIVST on ART initiation and the impact of HIVST on ART initiation. For instance, a study conducted in KZN, South Africa (Harichund et al., 2019), noted that individuals continue to value counseling support during testing, while one in Malawi (Dovel et al., 2023) reported lower ART initiation rates among the HIVST group. Other studies found no significant differences in ART initiation between HIVST and traditional testing groups (Neuman et al., 2021; Njau et al., 2019). Importantly, our results align with South African research (Mostert, 2022) and a systematic review and meta-analysis (Jamil et al., 2021), suggesting that HIVST drives increased testing uptake, particularly among infrequent testers and those in rural settings. In our sample, rural participants comprised a substantial majority (96%), underscoring HIVST’s potential to support testing and treatment engagement in resource-limited areas.

This study identifies gender inequality in the utilization of HIV testing and ART initiation. In bivariate analyses, gender differences are particularly pronounced, revealing that males are generally less likely to undergo HIV testing compared to females. This finding is consistent with past DREAM studies (Baisley et al., 2019). Although the adjusted model diminished the role of gender inequality, it points to the importance of social determinants in predicting HIV testing and ART initiation prevalence.

The findings of this study offer a clear application of Andersen’s Behavioral Model of Health Services Use, providing insight into healthcare disparities (Andersen, 1995). According to the model, healthcare access is influenced by predisposing factors (e.g., education level, rural residence), enabling resources (e.g., proximity to health facilities, participation in HIVST programs), and need factors (e.g., STI screening). In this context, rural residence, lower education levels, and limited access to HIVST services emerge as significant determinants, consistent with the model’s emphasis on structural and individual influences on healthcare utilization. Therefore, interventions aimed at increasing access to HIV testing and ART initiation must target these structural inequities, with HIVST offering a promising strategy to improve privacy and reduce stigma-related barriers.

Despite its valuable insights, the study has several limitations, including its reliance on self-reported data, which may have introduced biases, as participants may have under or over-reported their testing behaviors due to social desirability. Additionally, the study’s cross-sectional design limited the ability to establish causal relationships between the identified factors and the outcomes of HIV testing and ART initiation. The focus on a specific geographic area may have limited the generalizability of the findings to other regions with different socio-economic and spatial contexts. Moreover, although HIV testing and ART services in the study area are supported by the government and various partner organizations which might impact our results, the exact locations of these services were not known. Finally, not all types of HIV testing modalities were captured in the analysis.

## Conclusion

The study reveals significant spatial disparities in HIV testing and ART initiation in the uMkhanyakude District, KZN, South Africa, particularly highlighting the lower prevalence of ART initiation in rural settings. The findings suggest that HIVST can increase testing uptake, particularly in rural and under-resourced areas, and thereby improve healthcare engagement. This research addresses healthcare inequities by identifying key factors (education level below secondary school, rural residence, not participating in HIVST programs, STI screening, treatment programs, and distance from health facilities) and suggesting targeted interventions, such as HIVST, to improve testing rates and ART initiation. Since then we have evaluated a range of peer-led and community-based mobile sexual health clinic models to improve the uptake of HIV testing and prevention and care (Shahmanesh et al., 2021; Shahmanesh et al., 2024). Evaluating the effectiveness of specific interventions aimed at reducing geographic and socio-economic barriers could provide deeper insights into strategies for improving HIV service delivery in rural settings.

## Figures and Tables

**Table 1.** Baseline characteristics of the participants who were assessed for HIV testing and ART initiation among adolescents and youth in the uMkhanyakude District from 2017 to 2019 (n = 5352).

Variable	Categories	HIV testing (n = 5352)		ART initiation (n = 680)	
		n	Percentage	n	Percentage
Age		Median = 19	IQR:16, 24	Median = 25	IQR:20, 28
Sex	Female	2868	53.59%	451	66.32%
	Male	2484	46.41%	229	33.68%
Education attainment	Some secondary (up to Grade 12)	833	38.58%	239	48.58%
	Completed Grade 12	1107	51.27%	217	44.11%
	Tertiary	219	10.14%	36	7.32%
Residence	Rural	5187	96.92%	654	96.17%
	Urban	162	3.08%	26	3.83%
Participate in a facility-based HIV test	Yes	2178	46.68%	340	53.80%
	No	2488	53.32%	292	46.20%
Counseling before your last HIV testing	Yes	3641	90.55%	569	93.43%
	No	380	9.45%	40	6.57%
Participate in HIV self-testing	Yes	159	4.49%	30	5.87%
	No	3381	95.51%	481	94.13%
Participate in STI screening and treatment	Yes	523	12.55%	138	24.17%
	No	3645	87.45%	433	75.83%
Distance to health facilities	<3km	2421	45.68%	316	47.02%
	3–5km	919	17.34%	159	36.66%
	>5km	1960	36.98%	197	29.32%
Social media exposure	Use any social media	2332	76.21%	350	78.85%
	Don’t use	728	23.79%	94	21.17%

The sample size decreases for education attainment, participation in HIV self-testing, and social media exposure due to missing values.

**Table 2.** Associated factors of HIV testing among adolescents and youth in the uMkhanyakude District from 2017 to 2019.

Variable	Categories	Simple linear regression (β)	95% CI	Multivariable linear regression (β)	*p*-value	95% CI
Age in years	13–15	(ref)		(ref)		
	16–19	0.45	−1.05, 1.48	4.84	0.602	−13.43, 23.16
	20–24	1.51	−0.08, 3.03	3.52	0.700	−14.42, 21.48
	25–35	4.66	3.03, 6.15	4.41	0.630	−13.51, 22.32
Sex	Female	(ref)		(ref)		
	Male	−1.65	−2.63, −0.51	−0.77	0.295	−2.22, 0.67
Education	Some secondary (up to Grade 12)	(ref)		(ref)		
	Completed Grade 12	1.56	−0.37, 3.27	0.70	0.337	−0.73, 2.13
	Tertiary	9.6	6.93, 12.98	4.53	0.000	2.11, 6.88
Residence	Rural	−25.82	−26.85, −24.75	−31.14	0.000	−32.32, −29.65
	Urban	(ref)		(ref)		
Participate in facility-based HIV testing.	Yes	(ref)		(ref)		
	No	0.46	−0.67, 1.59	−0.99	0.135	−2.30, 0.31
Counseling before your last HIV testing	Yes	(ref)		(ref)		
	No	−0.49	−2.57, 1.59	−0.08	0.956	−2.93, 2.77
Participate in STI screening and treatment.	Yes	(ref)		(ref)		
	No	−3.67	−5.48, −1.82	−1.25	0.183	−2.89, 0.55
Distance to nearest health facilities	<3m	−6.01	−7.18, −4.86	−2.42	0.001	−3.88, −0.96
	3–5km	−0.21	−1.74, 1.30	2.11	0.031	0.16, 3.88
	>5km	(ref)		(ref)		
Social media exposure	Use any social media	(ref)		(ref)		
	Don’t use	−1.1	−2.69, 0.66	0.66	0.448	−1.04, 2.36

(ref) is referring to the reference categories

**Table 3.** Associated factors of ART initiation among adolescents and youth in the uMkhanyakude district, KZN, South Africa, from 2017 to 2019.

Variable	Categories	Simple linear regression (β)	95% CI	Multivariable linear regression(β)	*p*-value	95% CI
Age in year	13–15	(ref)		(Ref)		
	16–19	−0.07	−1.57, 1.42			
	20–24	−0.05	−1.52, 1.42			
	25–35	0.77	−0.61, 2.16	−0.52	0.655	−2.84, 1.79
Sex	Female	(ref)		(ref)		
	Male	−0.63	−1.30, 0.03	−0.94	0.094	−2.04, 0.16
Education	Some secondary (up to Grade 12)	(ref)		(ref)		
	Completed Grade 12	−0.62	−1.40, 0.15	−0.56	0.317	−1.67, 0.54
	Tertiary	1.04	−0.45, 2.52	−0.46	0.689	−2.68, 1.77
Residence	Rural	−5.31	−6.86, −13.66	−5.44	0.000	−7.38, −3.37
	Urban	(ref)		(ref)		
Participate in facility-based HIV testing.	Yes	(ref)		(ref)		
	No	−0.25	−0.92, 0.41	−0.59	0.263	−1.63, 0.44
Participate in HIV self-testing	Yes	2.64	1.11, 4.19	3.21	0.015	0.64, 5.80
	No	(ref)		(ref)		
Counseling before your last HIV testing	Yes	(ref)		(ref)		
	No	−0.35	−1.73, 1.03	0.46	0.753	−2.42, 3.34
Participate in STI screening and treatment.	Yes	(ref)		(ref)		
	No	−0.98	−1.79, −0.16	−1.22	0.044	−2.44 −0.03
Distance to nearest health facility	<3m	−1.05	−1.86, −0.38	0.06	0.911	−1.13, 1.27
	3–5km	−0.14	−1.02, 0.73	1.1	0.130	−0.32, 2.54
	>5km	(ref)		(ref)		
Social media exposure	Use any social media	(ref)		(ref)		
	Don’t use	−0.80	−1.77, 0.16	−1.23	0.075	−2.53, 0.12

(ref) is referring to the reference categories

## Data Availability

The datasets generated and/or analyzed during the current study are available in the AHRI repository and will be made accessible prior to publication.
